# Dimensions and forms of artefacts in 1.5 T and 3 T MRI caused by cochlear implants

**DOI:** 10.1038/s41598-022-08988-2

**Published:** 2022-03-22

**Authors:** Timo M. Gottfried, Daniel Dejaco, Natalie Fischer, Veronika Innerhofer, Lejo Johnson Chacko, Gerlig Widmann, Christian Kremser, Herbert Riechelmann, Joachim Schmutzhard

**Affiliations:** 1 Department of Otorhinolaryngology, Head and Neck Surgery, Medical University of Innsbruck, Anichstr. 35, 6020 Innsbruck, Tyrol Austria; 2 Department of Radiology, Medical University of Innsbruck, Anichstr. 35, 6020 Innsbruck, Tyrol Austria

**Keywords:** Neuroscience, Auditory system, Cochlea

## Abstract

Cochlear implantation is a standard treatment option due to expanding indications. Cranial magnetic resonance imaging (cMRI) has become a widespread diagnostic tool. Therefore, an increased number of cochlear implant (CI) users are undergoing cMRI scans. This study aimed to investigate the issue of the CI magnet impacting MRI quality and artifacts. 1.5 T and 3 T MRI scans with 4 defined sequences (T2-TSE, T2-TIRM, T1-3D-MPRAGE, and TDI) were performed on a phantom with a CI (SYNCHRONY System by MED-EL Austria) in place. The resulting MRI artifacts were retrospectively compared to MRI artifacts observed in patients with a CI. All images were transferred to AMIRA and visualized by manual segmentation. Usable image quality was achieved in three sequences (T2-TSE, T2-TIRM and T1-mprage). Observed artifacts differed in shape and size depending on the sequence. Maximum diameters of signal void areas ranged from 58 × 108 × 98 mm to 127 × 123 × 153 mm. Image distortions were larger. MRI artifacts caused by the SYNCHRONY system are asymmetric with varying shape, depending on the sequence. The phantom artefacts are similar to those in CI users. Considering the observed asymmetry, the hypothesis of varying implantation locations resulting in varying positions of the signal void area needs to be further investigated.

## Introduction

Cochlear implantation has developed into a standard treatment option for patients with severe to profound hearing loss^1,2^. The number of patients treated with a cochlear implant (CI) rose from approx. 110,000 patients in 2008^1^ to over 400,000 patients in 2017^3^ worldwide. This rapid development was driven by an extension of the CI indication criteria which now cover all age groups ranging from newborns to elderly people with hearing loss^4^. The rising number of patients and the higher age of CI recipients increase the likeliness that CI users will require cranial imaging modalities after cochlear implantation.

Cranial magnetic resonance imaging (cMRI) is frequently used in the diagnostic work-up of neurological^5,6^ and otologic diseases^7,8^. The increasing indications for cMRI, better availability, and lower examination cost have led to an increased implementation of cMRI worldwide^9^.

As a consequence, the number of cMRI scans in CI users is also increasing. MRI scans in this cohort are still subject to certain limitations, e.g. pain, heat development, demagnetization, and dislocation of the inner magnet^10–13^. The safety limitations are directly linked to the magnetic field strength measured in Tesla (T). Therefore, MRI-compatible CI systems approved for up to 3 T MRI scans were developed by different manufacturers^14,15^.

Nonetheless, also these innovative CIs contain metallic and magnetic components which cause artifacts in cMRI scans and thus decrease their diagnostic value. The artifacts vary in size and shape depending on magnetic field strength, cMRI sequence, and position of the internal CI magnet^16–18^.

At 3 T, artifacts caused by the CI have been shown to affect image quality of the nearby brain regions depending on the chosen sequence. Majdani et al. observed additional periodic shadowing especially in T2-weighted sequences in 3 T cMRI performed with one phantom and three cadaveric specimens. The authors concluded that these artifacts reduce the likeliness of accurate diagnosis of brain lesions on the ipsilateral side^18^.

In a retrospective chart review in 2016, Sharon et al. evaluated 57 MRI brain scans of CI users. This analysis revealed several factors influencing the image quality with a CI magnet in situ. One main factor influencing the size and shape of the artifact was the MRI sequence used^19^.

This observation was further scrutinized by Edmonson et al. Their findings support the hypothesis of a correlation between the expected artifact and the applied MRI sequences^20^. Furthermore a non-symmetric shape of these artifacts has been discussed^19^. However, these three-dimensional shapes have yet to be described depending on the MRI sequence and magnetic field strength applied.

The aim of this study was to visualize and quantify artifacts caused by a specific cochlear implant system (MED-EL SYNCHRONY) in frequently used cMRI sequences in 1.5 T and 3 T magnetic fields. Artifact visualization and size measurement was performed using a specific MRI phantom.

## Materials and methods

The 3 T images were acquired using a whole body MRI scanner (MAGNETOM SKYRA, Siemens, Erlangen; Germany); the 1.5 images were acquired using a MAGNETOM AVANTO (Siemens, Erlangen; Germany).

The sequences were chosen according to the in-house protocol for cerebellopontine angle imaging including: (1) T2-weighted turbo spin echo (T2-TSE), (2) T2-weighted Turbo inversion recovery with magnitude reconstruction (T2-TIRM) and (3) T1-weighted 3D-magnetization-prepared rapid gradient echo (3D-MPRAGE)^21^, (4) diffusion tensor imaging sequence (DTI). Sequence parameters are listed in Table 1.

**Table 1:** Image specifications.

	T2-TSE	T2-TIRM	3D-MPRAGE	DTI
TR (MS)	5800	8000	7.8	7000
TE (MS)	95	87	2.94	95
TI (MS)	–	2370	1100	–
Flip angle	90°/150°	90°/150°	8°	90
Echo train length	16	16	1	64
Pixel bandwidth (Hz/Px)	220	250	250	1500
Acquisition matrix	218 × 384	175 × 320	187 × 256	64 × 128
Reconstructed matrix	312 × 384	250 × 320	208 × 256	256 × 256
Field of view (mm)	178 × 220	171 × 220	178 × 220	220 × 220
Slice thickness (mm)	2	3	1	3
Gap between slices (mm)	0	0.6	0	0,3
Number of slices	75	45	176	43
Number of concatinations	3	2	1	1
Parallel imaging mode	GRAPPA	–	GRAPPA	GRAPPA
Parallel imaging factor	2	–	2	2
Number of diffusion encoding directions	–	–	–	20
B-value	–	–	–	1000
Voxel size (mm)	0.573 × 0.573 × 2	0.688 × 0.688 × 3	0.859 × 0.859 × 1	0.859 × 0.859 × 3
Acquisition time	3 × 54.40 s	2 × 1 min 20 s	3 min 36 s	2 min 50 s

TR (ms): Repetition time (in milliseconds), TE: Echo time, TI: Inversion time, Hz/Px: Herz per pixel, mm: millimeter.

MRI with the above sequences was performed using a special MRI phantom which had been prepared with a MED-EL SYNCHRONY CI. This study is limited to devices implanted in our center. Furthermore, retrospective analysis of MRI images of CI users from the local database was performed. The artifacts were visualized as 3D models to enable exact metric measurement and 3D modeling.

### MRI phantom

**Figure 1:**
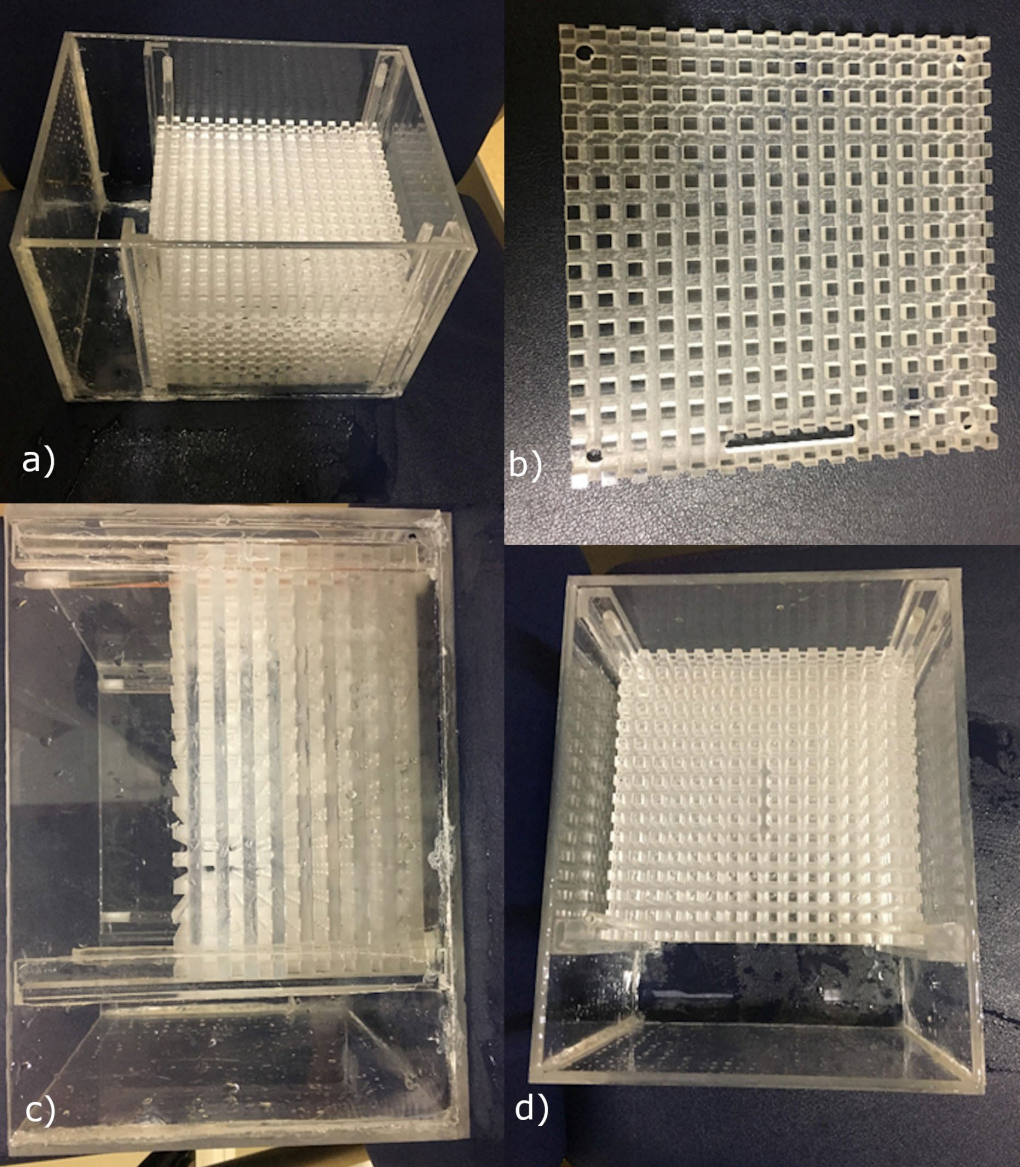
(**a**–**d**) The used model. In a plexiglass cube (**a**, **c**, **d**) multiple layers of 10-mm-thick Plexiglas plates (**b**) with 5 × 5 mm grids were stacked on top of each other.

The Phantom was used in accordance with Majdani O’s study from 2009^18^. 10-mm-thick Plexiglas plates with 5 × 5 mm grids were stacked on top of each other. The grids on each layer were placed on top of each other, so that each grid element formed a 5 × 5 × 5 mm cube resulting in a total volume of 17cmx17 cm. This 3D grid allowed a quantification of the artifacts and distortions caused by a MED-EL SYNCHRONY CI (Fig. 1).

Different experiment setups were conducted. First, the CI was placed on a lateral location, simulating a real device implanted on a human head. Second, the individual components of a CI (magnet and implant without magnet) and the complete device were placed in the center of the phantom to investigate the full dimensions of the artifact and which part of the CI is responsible for it.

To obtain an MRI signal, the phantom was filled with 3 L of 0.9% saline solution. For the scans the Cochlea Implant as immersed (Fig. 1). The prepared phantom was then placed in the standard 20-channel head coil of the MRI scanner and imaging using the sequences listed above was performed.

### Retrospective MRI images

Starting with 2013 a retrospective search of the existing hospital MRI database for cMRI images with CIs was performed and four patients were included matching our requirements in sequences taken and tesla strength used. No additional scans were performed because of this study. After defining critical anatomic landmarks, these images were evaluated by an in-house radiologist for their quality and readability with special regard to the above listed sequences.

All procedures performed in studies involving human participants were in accordance with the ethical standards of the institutional and/or national research committee and with the 1964 Helsinki Declaration and its later amendments or comparable ethical standards. Informed consent for the scientific use of performed images was obtained from all individual participants included in the study. *Because of the retrospective character of the study, the Ethics Committee of the Medical University of Innsbruck certified that no ethics committee approval is required under Austrian law.*

### Data visualization

**Figure 2:**
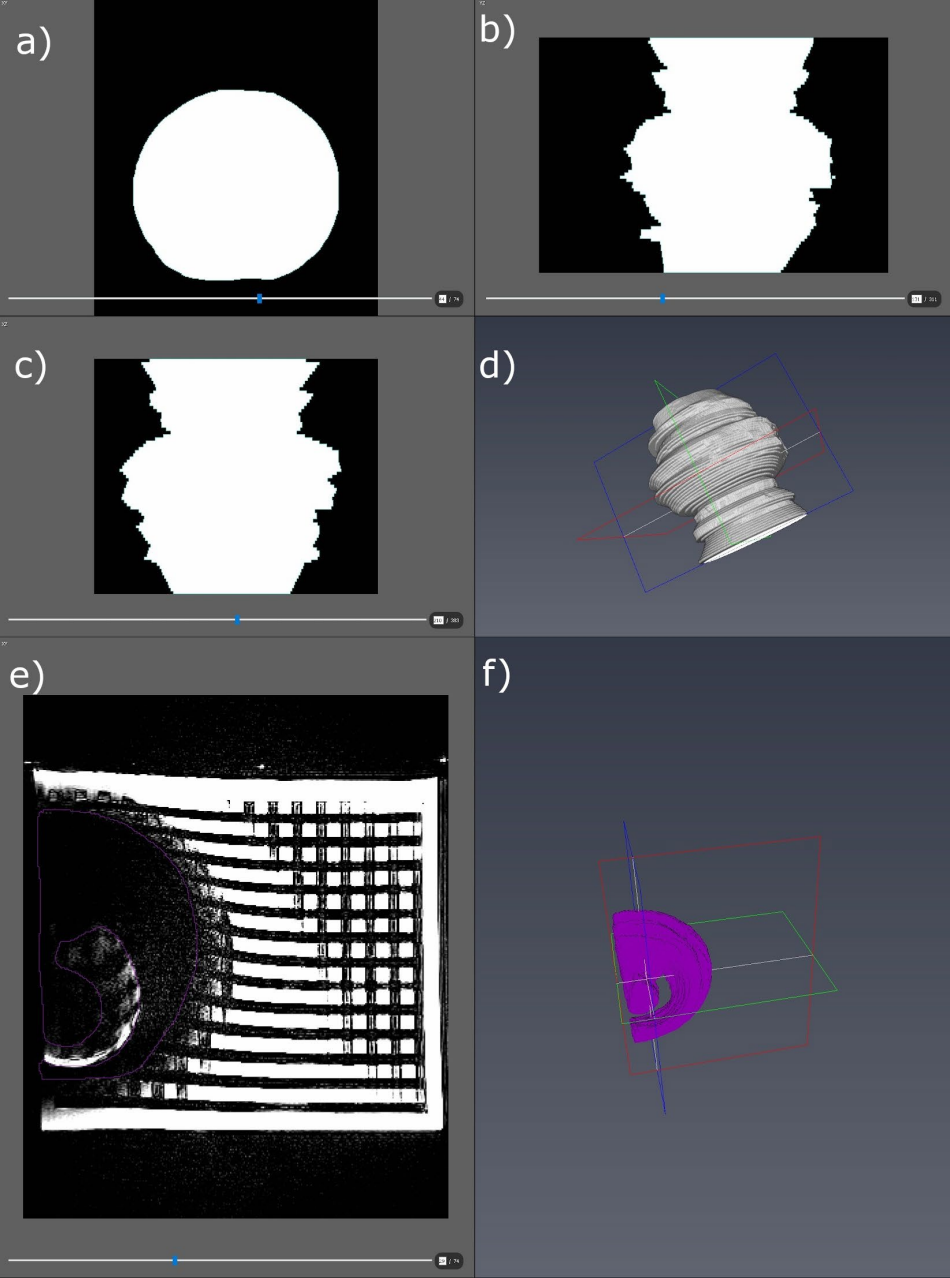
The segmentation process using AMIRA software. Insert (**a**–**c**) depicts the 2-dimensional view which has been marked by hand. In (**d**) the 3-dimensional composition gathered by (**a**–**c**) is visualized. Picture (**e**) shows a T2-TSE sequence in the segmentation process. The signal void area has been marked by hand in pink. Insert (**f**) visualizes the resulting 3-D image.

DICOM data from the phantom examinations and the retrospective search were transferred into AMIRA (Thermo-Fisher Scientific-Fei Visualization Science Group, Méignac Cédex, France). This software contains tools for 3D rendering, measurement, and defining (Fig. 2).

Using this program, each slice of the MRI scans was manually segmented twice, following Glueckert et al.^22^, creating a 3D model of (a) the signal void areas and (b) areas of distortion for each individual sequence.

Finally, the dimensions of these 3D models were measured using AMIRA and transferred to MICROSOFT Excel files.

## Results

The following MRI sequences were used with the phantom: T2-TSE, T2-TIRM, 3D-MPRAGE and DTI. The acquired images were evaluated for possible usability. All diffusionweighted images were excluded due to poor quality and classified as not readable.

T2-TSE, T2-TIRM and 3D-MPRAGE sequences were assessed as readable, providing partially usable information. The images obtained with these sequences were manually segmented using the AMIRA visualization program as described above.

### T2-weighted turbo spin echo

**Figure 3:**
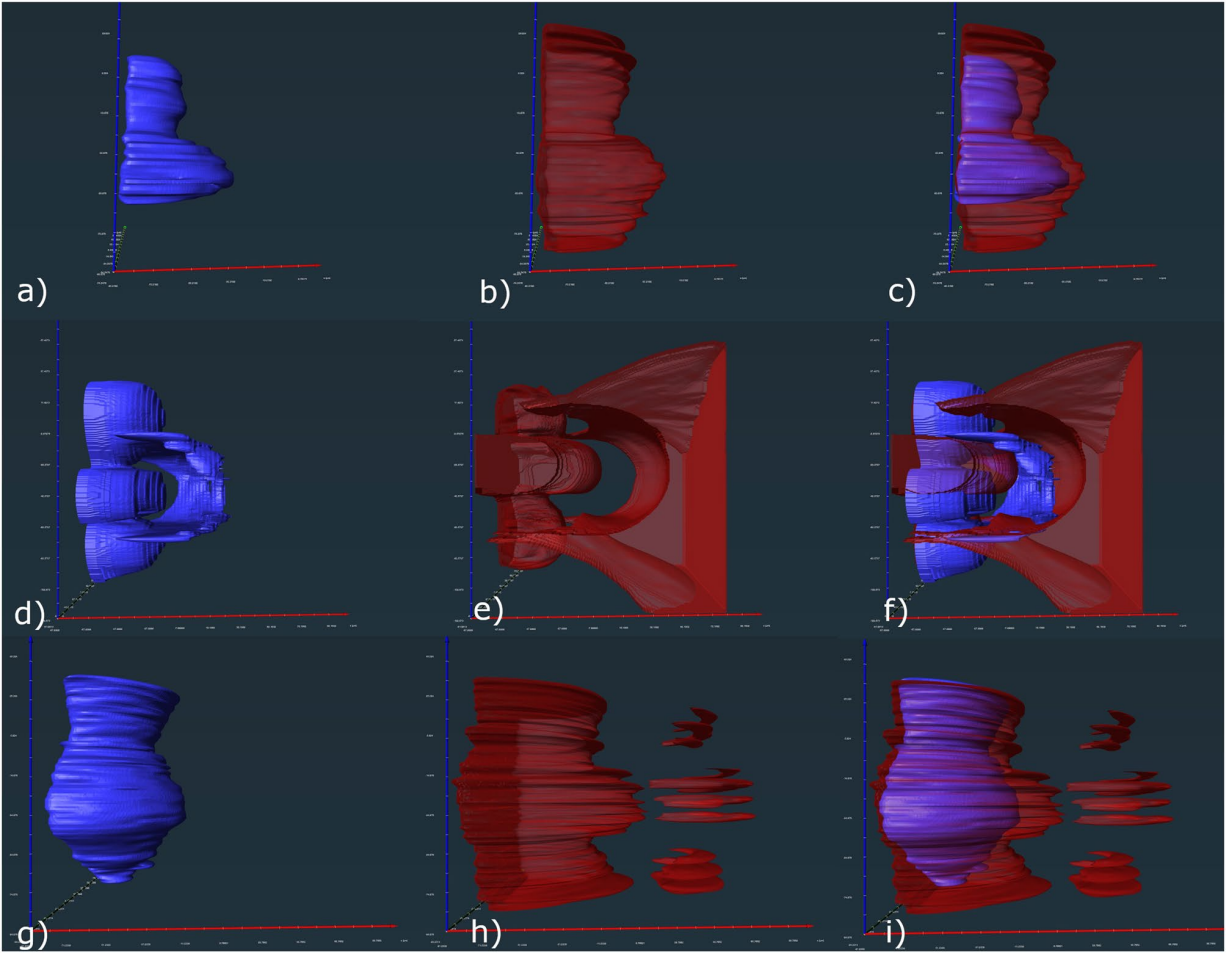
The signal void and distorted areas caused by a MED-EL SYNCHRONY CI laterally placed on the phantom for each of the included sequences. Inserts (**a**–**c**) show the results for T2-TSE. The signal void area is visualized in a, the distorted area in (**b**). Picture (**c**) shows the combination of (**a**) and (**b**). the pink and blue coloring shows results of two different segmentations, which appear to be identical. In picture (**d**–**f**) the T2-tirm sequence is presented with the signal void area in (**d**), the distorted area in (**e**) and the combination in (**f**). T1-mp_rage sequence is shown in (**g**–**i**) with the signal void area in (**g**), the distorted area in (**h**) and the combination in (**i**).

In the phantom with a laterally placed CI, the T2-TSE sequence in 3 T MRI resulted in signalvoid areas with the following measurements: 66 mm in coronary, 117 mm in sagittal and 98 mm in axial dimension (Table 2). The extent of the distorted area in a 3 T magnetic field was 94 × 135 × 145 mm (Table 2) (Fig. 3).

**Table 2:** Maximum extent of signal void areas in mm and distorted areas in mm.

Sequences	Coronary	Axial	Sagittal
**Phantom 3 T signal void area**			
T2-TSE	66	117	98
T2-Tirm	121	117	136
T1 mp_rage	88	122	140
**Patient 1.5 T signal void area**			
T2-TSE	58	107	92
T2-Tirm	127	123	153
T1 mp_rage	74	155	115
**Patient 3 T signal void area**			
T2-TSE	63	103	121
T2-Tirm	121	117	136
T1 mp_rage	62	114	83
**Phantom 3 T distorted**			
T2-TSE	94	135	145
T1 mp_rage	174	154	140
**Patient 1.5 T distorted**			
T2-TSE	66	125	103
T1 mp_rage	85	161	110
**Patient 3 T distorted**			
T2-TSE	74	146	102
T1 mp_rage	120	200	142

**Figure 4:**
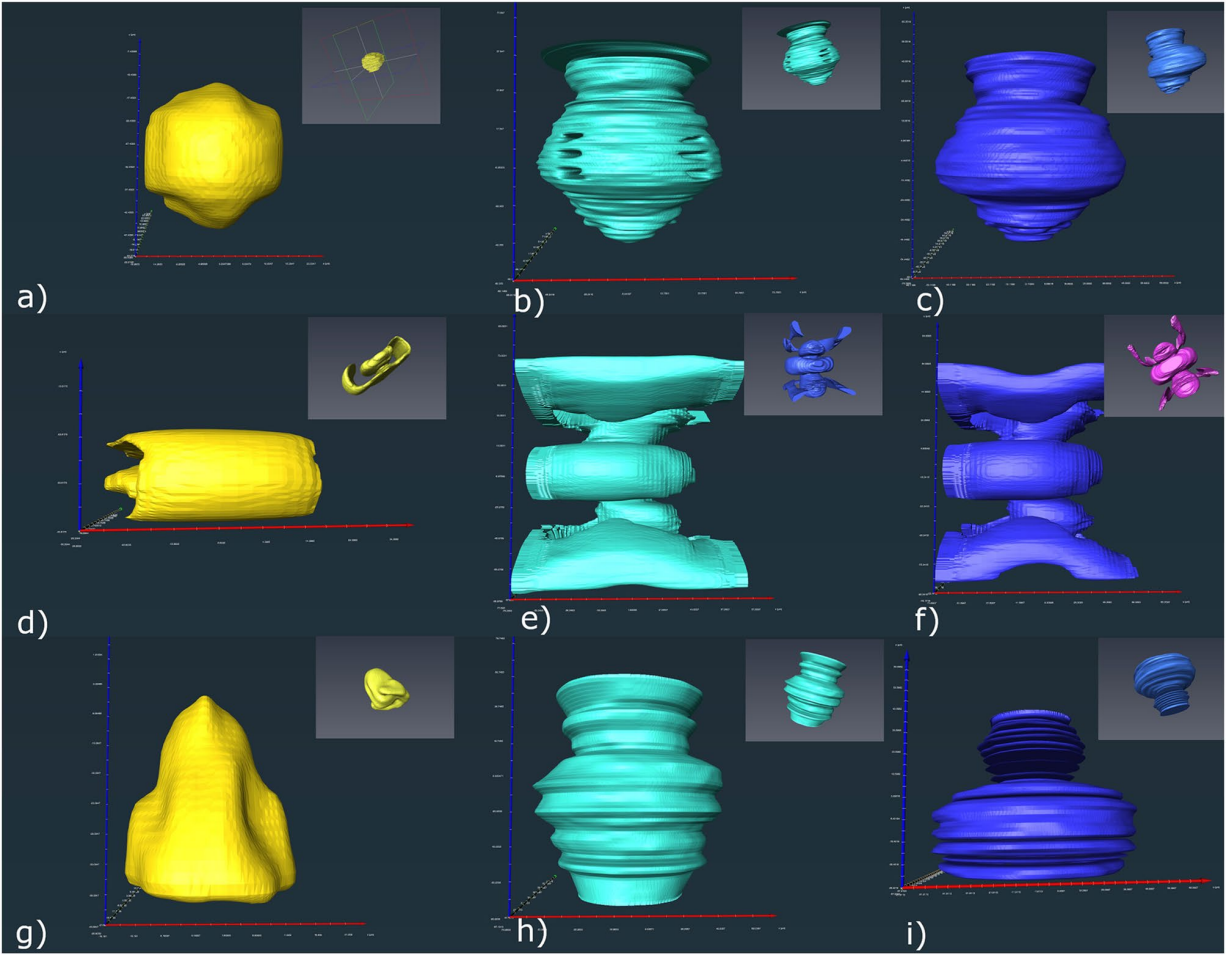
The 3D shapes of artifacts caused by the individual components—casing, magnet and the assembled system. Insert (**a**–**c**) visualize the T1-mp_rage sequence. The signal void area caused by the casing is seen in (**a**, **b**) visualizes the signal void area caused by the magnet and (**c**) the signal void area caused by the assembled system. In (**d**–**f**) the T2-tirm sequence is presented—(**d**) casing, (**e**) magnet and (**f**) assembled system. The pictures (**g**–**i**) show the results for the T2-TSE sequence with (**g**) representing the casing, (**h**) the magnet and (**i**) the assembled system.

The measurements of the individual CI components placed in the center of the phantom model (Fig. 4) with T2-TSE sequences resulted in a signal-void artifact of 26 × 46 × 43 mm for the casing, 121 × 97 × 122 mm for the magnet and 123 × 96 × 128 mm for the complete CI (Table 3). The maximum extent of distorted areas was 139 × 148 × 135 mm for the sole magnet, 50 × 66 × 60 mm for the casing, and 151 × 128 × 146 mm for the complete CI (Fig. 4).

**Table 3:** Dimensions of the signal void area areas individual components in mm.

Sequences	Coronary	Axial	Sagittal
**Magnet 3 T signal void area**			
T2-TSE	121	97	122
T2-Tirm	159	170	152
T1 mp_rage	129	134	135
**Casing 3 T signal void area**			
T2-TSE	26	46	43
T2-Tirm	66	26	75
T1 mp_rage	38	44	51
**CI 3 Tesla signal void area**			
T2-TSE	123	96	128
T2-Tirm	151	172	150
T1 mp_rage	127	127	129

**Figure 5:**
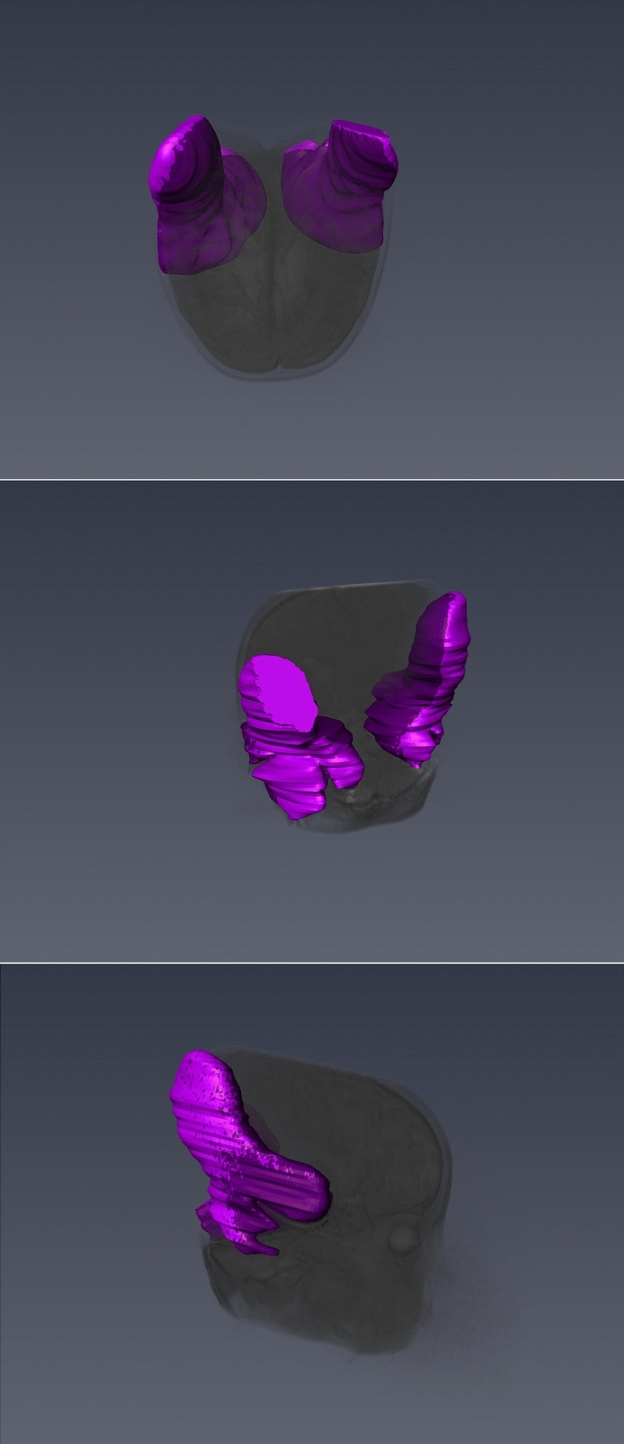
The position of the artifact in correlation to the human head. The example shows the typical T2-TSE sequence artifact in a 1.5 T setting of a bilaterally implanted patient. The orientation of the artifacts exhibits a mirror symmetry along a sagittal plane in the middle of the head.

Segmented signal void artifacts in the retrospectively analyzed MRI images of CI users were 58 × 107 × 92 mm in 1.5 T and 63 × 103 × 121 mm in 3 T fields. Distorted areas extended to a maximum of 66 × 125 × 103 mm using 1.5 T and 74 × 146 × 102 mm using 3 T (Fig. 5).

### T2-weighted turbo inversion recovery magnitude

Applying 3 T TIRM-Imaging on the phantom with a laterally placed CI, signal void areas measured 121 × 117 × 136 mm (Fig. 3).

According to the T2-TSE protocol, the CI and its components were placed individually in the center of the model and 3 T TIRM-imaging was conducted for each of them. Their segmented artifacts showed a maximum dimension of 151 × 172 × 150 mm (CI), 159 × 170 × 152 mm (magnet) and 66 × 26 × 75 mm (casing) (Fig. 4).

Signal void areas in the retrospectively segmented CI user data measured 127 × 123 × 153 mm in a 1.5 T and 121 × 117 × 136 in a 3 T magnetic field (Fig. 5).

T2-TIRM sequences generated distorted areas of a greater extent than the used model and were therefore excluded from this study as they were unmeasurable.

### T1-weighted 3D magnetization-prepared rapid gradient echo sequence

Applying the T1-3D-MPRAGE sequence on the phantom in 3 T condition, the artifact measured 88 × 122 × 140 mm in the signal-void area and 174 × 154 × 140 mm in the distorted area (Fig. 3).

The individual components caused artifacts with a size of 127 × 127 × 129 mm for the complete CI, 129 × 134 × 135 mm for the magnet, and 38 × 44 × 51 mm for the casing. Distorted areas measured 150 × 139 × 144 mm (CI) and 137 × 134 × 139 mm (magnet) (Fig. 4).

The retrospective data showed artifacts of 74 × 155 × 115 mm at 1.5 T and 62 × 114 × 83 mm at 3 T. The maximum extent of distorted areas was 85 × 161 × 110 mm at 1.5 T and 120 × 200 × 142 mm at 3 T (Fig. 5).

**Figure 6:**
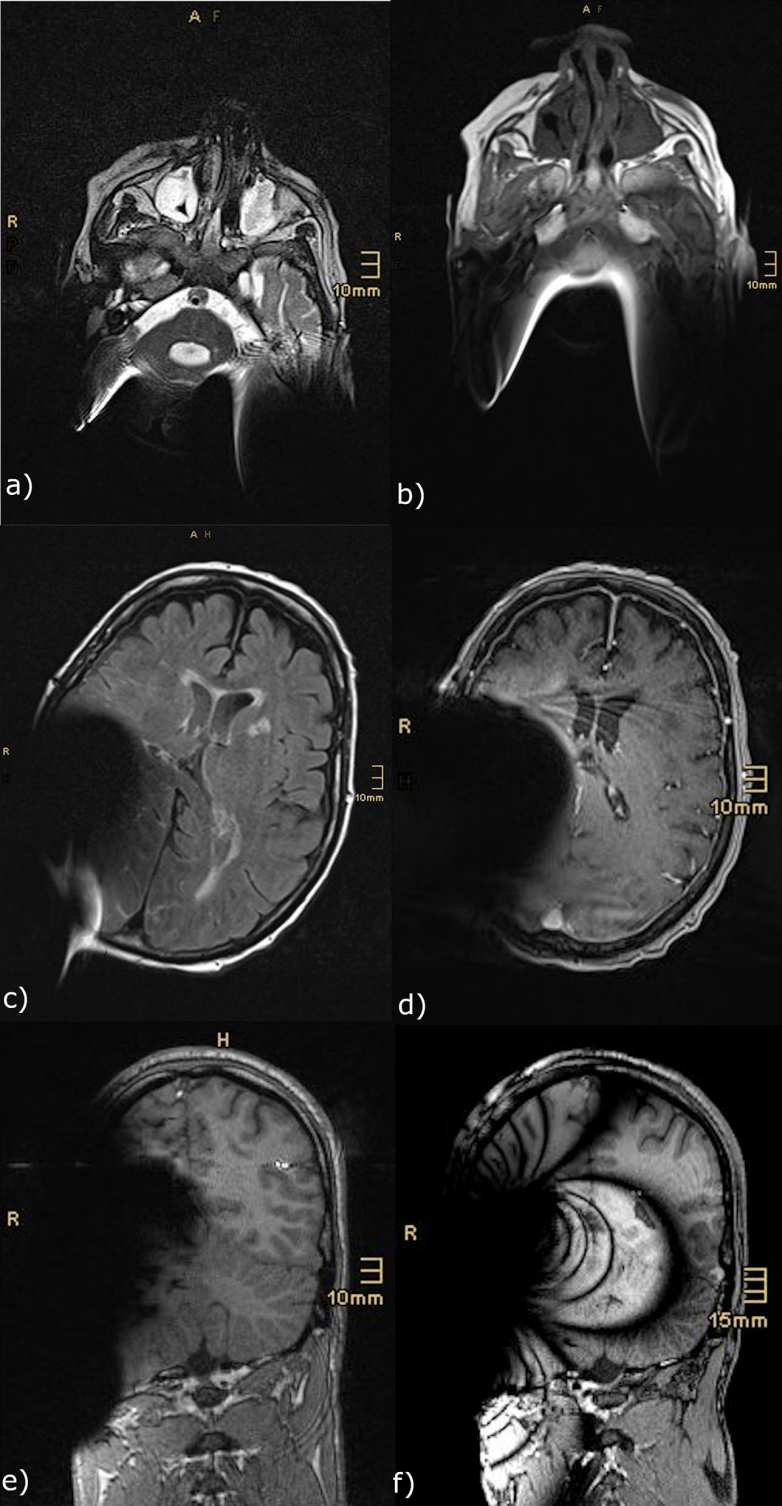
Examples of the retrospective data used in this study. The pictures (**a**, **c**) present different layers of 1.5 T T2 TSE sequences done in a patient with a cochlear implant on (**a**) both sides and (**c**) the right side. Pictures (**b**) and (**d**) show a comparable layer of the same patient done in a T1-mp_rage sequence. Picture e) visualize exemplary an artefact caused by a CI on the right side in a 1.5 T MRI (T1-mp_rage), while picture (**f**) represents nearly the same layer of an image done in a 3 T device.

Furthermore, the data was reviewed by an in-house radiologist to evaluate the use of image quality and readability. Focus was the clinical use and assessability of important anatomic structures like medulla oblongata, inner ear, cerebellar bridge angle, as well as cerebrospinal fluid space and the cerebellum. In general, all T2 TSE sequences provided a distinctly better visualization of the inner head compartments compared to the T1-mp_rage sequences (Fig. 6a,b). Only the cerebrospinal fluid spaces were comparable in their radiological value (Fig. 6c,d). T2-TIRM sequenced images provided nearly the same image quality and visualization of critical anatomic landmarks as T2 TSE sequences. Merely the assessment of the inner ear canal was clearly better in T2 TSE sequences.

## Discussion

All types of implants with metallic and magnetic components are well known to cause artifacts in MRI scans. The potential of CIs to cause artifacts in MRI scans has been described in multiple publications^17–20^. In this study, the artifacts caused by the SYNCHRONY CI system (MED-EL, Austria) were visualized using MRI phantom trial images and MRI images of CI users. The observed artifacts differed in shape, size, and symmetry depending on the sequences used.

When applying a standard cMRI protocol, a primary differentiation between readable and unreadable sequences needs to be made. All diffusion sequences (ADC, tracer-weighted, and tensor diffusion-weighted) were unreadable with the magnet in place. This observation was previously published by Edmonson et al. who described diffusion-weighted sequences with significant geometric distortion and intravoxel dephasing^20^. As concluded by Edmonson et al. and also confirmed in the present study, the usability and necessity of diffusion-weighted images with the CI magnet in place must be scrutinized and might be dispensable.

T2 TSE, T1 MP_RAGE and T2 TIRM were classified as partly readable with varying size, shape, and quality of the artifact. A signal-void area, which comprised a black unreadable area close to the magnet, could be distinguished from a distortion area with partly distorted but readable visual information.

Different shapes depending on the used sequence and magnet strength were observed in the phantom pictures and real patient pictures. The T2 TSE sequence visualized a chalice-like signal-void area with a surrounding distortion area. The T1 MP-RAGE sequence visualized a spinner-like signal-void area with a small distal tip and proximal widening and a surrounding distortion area. The T2 TIRM sequence did not show a distortion area. The signal void region in the cross section had a flower-like structure (Fig. 3a–i).

The above described gained knowledge of the various shapes of the artifacts, will allow a more selective application of MRI sequences. This can result in a future reduction of cost and time. For example knowing the spinner-like shape of a T1 MP-RAGE artefact helps in the positioning of the CI intraoperatively. A more apical position of the implant moves the larger superior diameter to “less” important parts of the brain, enabling an evaluation of the crucial brainstem. A similar effect is achieved in the T2 TSE sequence with the chalice formed artefact.

These asymmetries seem to depend on the special and unique structure of the CI itself. The artifacts were identical in the phantom pictures and the patient pictures (Figs. 3, 4). The different shapes, however, may provide opportunities regarding their location in relation to the different anatomical structures. It should be possible to influence their location by changing the implantation angle or distance to the inner ear. This indicates that it could be possible to position the CI in a way that favors MRI scans of important intracerebral structures, e.g., the brainstem or the corpus callosum.

Ingo Todt and colleagues assessed this hypothesis to some extent. In a recent study, Todt et al. tested different implant locations with regard to the resulting artefact. In this approach the angle between the implanted magnet and the nasion-external auditory canal, and variable distances of the implant to the inner ear were evaluated. Then imaged at 3 T^23^. Their results show a clearly better visualization of the inner ear canal and cerebral structures with the CI being located farther away (9 cm) and with a higher insertion angle (120°). This further verifies the assumption above. In 2018, Dirk Schröder and colleagues carried out a study with a very similar protocol using 1.5 MRI. Dewey and coworkers confirmed this finding in a 3 T magnetic field. Their results correspond with the previous studies^24,25^. However, in all three study protocols they fixed the CI onto the head with a tight dressing and did not proceed an implantation. The workgroup around Pietro Canzi took a first step into this direction by performing the first cadaver study evaluating MRI-induced artifacts in actual heads with a CI. They implanted ULTRA 3D CI (ADVANVED BIONOICS, Stafä, Switzerland) with Slim J electrode arrays in three human cadaveric heads and applied 1.5 T scans investigating the assessability of different cerebral structures^26^. However no different implantation angles or distances were addressed and only 1.5 T scans were investigated. Further studies are required to confirm this hypothesis to change the implant location. In a recent follow up publication, Ay and colleagues investigated the alteration of a CI artefact in a 3 T MRI in different head positions of the patient. They found, that in fact the artefact can be influenced by applying either a reclinated or anteflexated head position and also concluded, that a chin-to-chest position enhances the quality of MR Images even in an unfavourable magnet-to-external auditory canal distance or angle^27^. This recent finding combined with a changed implantation distance and angle as well as the knowledge of the exact 3D proportions of the resulting artefacts could considerably improve MRI quality in CI patients.

Applying MR imaging of cochlear implants at 1.5 T and 3 T, this study revealed smaller artifacts in 1.5 T examinations than in 3.0 T scans (Fig. 6e–f). Using T2-weighted TSE sequences, artifacts were 7–10 mm smaller in the 1.5 T examinations than in the 3 T examinations. Using T1-weighted multiplanar sequences, the difference between 1.5 and 3 T goes up from 12 to 43 mm. Based on this observation, 1.5 T examinations should be preferred when imaging the head with a CI magnet in place to enable better visualization of the central brain structures.

This differs from the results Omid Majdani and colleagues (2009) found in their trial using the same study modalities as in the present study with prior MED-EL implant systems. They concluded that the artifacts produced in 1.5 T and 3 T MRI scans are about the same size and have no significant variance^18^. The difference between the outcomes of the study and Majdani’s et al.’s study can be explained with the further developed visualization technique applied in the present study.

In addition to the described magnet strength depending size difference discussed above an MRI sequence depending size variance can be observed as well. The T2-weighted turbo spin echo sequences produce significant smaller artifacts than T1-weighted mp_rage sequences. Therefore, the anatomical T2 TSE modality seems to be a favorable choice for anatomical cMRI examinations with the CI magnet in place. This finding matches the results of a previously published paper by Omid Majdani in 2008 regarding artifacts caused by CIs in 3 T MRI scans^18^. However, the maximum dimensions of the artifacts visualized in this study are up to 10 mm larger with the SYNCHRONY CI than with the PULSAR CI used in the 2008 publication. A possible explanation could be the different composition of the magnets. The PULSAR CI magnet consists of a samarium-cobalt whereas the SYNCHRONY CI consists of a neodymium iron boron mixture. In addition, shear force direction differs between the two magnets. However, the magnetic field strength is identical in both CI models. Additionally, both studies are a decade apart and therefore image quality and the number of images per unit may differ due to technical improvement of MRI scans. This could possibly be another reason for the varying results. This topic has been further evaluated by Todt et al. They addressed different magnets in various CI models and their resulting artefacts in MRI scans in an in vivo measurement with three different cochlear implant magnet systems—two with a 3D rotatable cylindrical bipolar implant magnet (AB 3D, ADVANVED BIONOICS, Stafä, Switzerland and MED EL SYNCHRONY, Innsbruck, Austria) and one first generation model magnet (OTICON ZTI, Vallauris, France)^28^. Although they found no major difference in maximum artefact sizes between the used magnet systems, a direct comparison to the PULSAR CI was not performed.

Defining which part of a CI device is responsible for the measured artefacts additional investigations were conducted. The magnet within a rubber casing dummy was placed in the center of the phantom and all sequences described were applied. This was repeated with the CI casing without the magnet and the whole CI device. The measurements of the different phantom MRI scans performed revealed the expected results. The magnet is responsible for nearly the entire artifact caused by a CI in MRI scans. As depicted in Table 2, the casing alone generates an artifact of 2.5 to 5 cm in maximum diameter whereas the magnet itself produces artifacts of up to 15 cm. Artifacts solely produced by the magnet led to the typical shapes described above, depending on the sequence applied. Whereas performing those sequences on just the casing resulted in symmetrical artifacts with no comparability to any shape described above.

These results support an experimental human cadaver study by Franca Wagner and colleagues in 2015 showing that the removal of the internal magnet can significantly reduce the size of artifacts caused by a CI in MRI scans^29^. In one retrospective case, a patient had a CI implanted on both sides; and as depicted in Fig. 5, the shape and position of the resulting artifact does not depend on the side of surgery. In fact, both artifacts could be mirrored about a sagittal plane between the brain hemispheres. This means that the maximum and minimum extent of the artifact on each side are located at the same height and position of the head and therefore mask the same anatomic structures. The main reason for the caused unreadable area is the magnet as mentioned above. One explanation for this might be that the magnet of the MED-EL SYNCHRONY is rotatable and can therefore reposition in the magnetic field. There are no recent studies to support this hypothesis. Further investigation needs to be conducted to verify this theory.

## Conclusion

Artifacts in MRI scans caused by the SYNCHRONY CI system vary in shape and size depending on the sequences applied. Reducing the MRI scan to readable sequences can save time and costs. Furthermore, the artifact shape is asymmetrical inside human head tissue. With this observed asymmetry, the hypothesis of varying implantation locations resulting in varying positions of the signal void area needs to be further investigated. It seems possible to visualize intracerebral structures by changing the implantation area or insertion angle. Further studies are needed to verify this conclusion.

## Data Availability

All data regarding this study is available.

## Abbreviations

CI: Cochlea implant
cMRI: Cranial magnetic resonance imaging
T: Tesla
T2_TSE: T2-weighted turbo spin echo
T2-TIRM: T2-weighted turbo inversion recovery with magnitude reconstruction
T1_MPRAGE: T1-weighted 3D magnetization prepared rapid gradient echo
DTI: Diffusion tensor imaging
